# Role of Mesenchymal Stem/Stromal Cells in Coagulation

**DOI:** 10.3390/ijms231810393

**Published:** 2022-09-08

**Authors:** Raquel Guillamat-Prats

**Affiliations:** Lung Immunity Translational Research Group in Respiratory Diseases, Germans Trias i Pujol Research Institute (IGTP), 08914 Badalona, Spain; rguillamat@igtp.cat; Tel.: +34-93-554-3050

**Keywords:** coagulation, mesenchymal stem/stromal cells, platelets, tissue factor, phosphatidylserine, inflammation

## Abstract

Mesenchymal stem/stromal cells (MSCs) are widely used in disease models in order to control several phases in the response to injuries, immune reaction, wound healing, and regeneration. MSCs can act upon both the innate and adaptive immune systems and target a broad number of functions, such as the secretion of cytokines, proteolytic enzymes, angiogenic factors, and the regulating of cell proliferation and survival. The role of MSCs in coagulation has been less studied. This review evaluates the properties and main functions of MSCs in coagulation. MSCs can regulate coagulation in a wide range of pathways. MSCs express and release tissue factors (TF), one of the key regulators of the extrinsic coagulation pathways; MSCs can trigger platelet production and contribute to platelet activation. Altogether, MSCs seem to have a pro-thrombotic role and their superior characterization prior to their administration is necessary in order to prevent adverse coagulation events.

## 1. Introduction

Mesenchymal stem/stromal cells (MSCs) are multipotent cells that can differentiate into several mesenchymal tissue lineages such as osteoblasts, chondrocytes, and adipocytes, but not hematopoietic stem cells [1,2,3,4,5]. As claimed by the International Society for Cell & Gene Therapy (ISCT), the abbreviation “MSC” refers to “stromal” and not to “stem”, unless stemness is proven. MSCs are resident in many tissues but can mostly be obtained from bone marrow, adipose tissue, and the umbilical cord, and the source will modify their characteristics [6]. MSCs can act upon both the innate and adaptive arms of the immune system and target a broad number of functions, such as the secretion of cytokines, proteolytic enzymes, and angiogenic factors, as well as regulating cell proliferation and survival [7]. Immunomodulatory, anti-inflammatory, anti-fibrotic, and anti-microbial effects have been linked to MSCs in many published pre-clinical studies. MSCs are fairly unique cells from an immunological standpoint and their properties enable the MSCs to escape recognition by the immune system, thereby modulating/mediating the T-cell, B-cell, NK, dendritic cell (DC), and macrophage functions [8,9,10,11].

MSCs have been used in disease models in order to control several phases in the response to injuries, immune reaction, wound healing, and regeneration. [12]. Furthermore, MSCs have been used to treat cardiac and pulmonary diseases, vascular and metabolic pathologies, and neurological disorders. MSCs exert their functions through several mechanisms such as cell to cell contact and cause a paracrine-derived effect by secreting the factors or the exosomes/microvesicles (EVs) into the microenvironment.

The role of MSCs in coagulation has been less studied, but in recent months, it has peaked the interest of many and a number of articles have been published, largely due to the importance of the clinical implications [5,13,14]. The sources and production processes will lead to different MSC-therapies that display varying levels of the procoagulant tissue factor (TFs) [6]. The TF is the major determinant of the cell product hemocompatibility and may negatively trigger an inflammatory systemic response under certain conditions. This review describes the properties and main functions of MSCs in coagulation. Coagulation and inflammation pathways have many nodes in common, and we examine how MSCs affect coagulation directly or indirectly.

## 2. Coagulation Factors and Inflammatory Linked Pathways

Coagulation and inflammation are highly cohesive systems; both are precisely regulated biological systems with extensive cross-talk that optimizes the organism’s response to any damage. An increased inflammation can trigger coagulation and that, sequentially, can augment inflammation [15]. The failure of the anticoagulant mechanisms to control the clotting procedure would increase the inflammatory process. In addition, the inflammatory mechanisms alter the hemostatic balance to promote the activation of coagulation, and it could produce intravascular coagulation or thrombosis.

It has been described that the inflammatory mediators can elevate the platelet count and reactivity, downregulate the natural anticoagulant mechanisms, initiate and propagate the coagulation response, and impair fibrinolysis. Correspondingly, clotting can increase the inflammatory response by releasing mediators from the platelets and activating cells that intensify the inflammatory responses.

The molecular and cellular nexuses between coagulation and inflammation are slowly being described, and here we summarized the most well-known and demonstrated interactions. The dysregulation of one or both systems impacts the entire balance, resulting in a pathological state with excessive inflammation or thrombosis.

### 2.1. Tissue Factor

**Figure 1:**
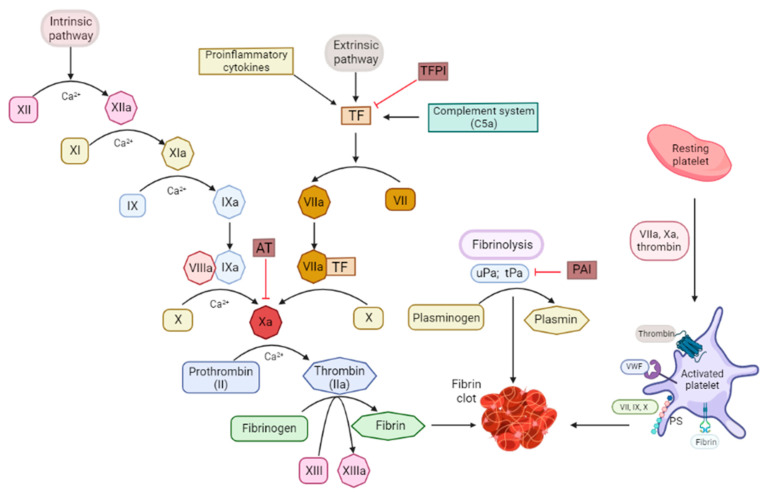
Schema of the intrinsic and extrinsic coagulation pathways and the fibrinolysis and platelet-coagulation interaction nodes. PAI: plasminogen activator inhibitor; PS: phosphatidylserine; TF: tissue factor; TFPI: tissue factor pathway inhibitor; tPA: tissue plasminogen activator; uPA: urokinase type-plasminogen activator; VWF: von Willebrand factor.

The tissue factor (TF), also called CD142, is one of the central nexuses between coagulation and inflammation. The TF is a type I integral transmembrane glycoprotein expressed by various cells. The TF is typically detected at high levels in the brain, skin, lungs, and placenta as well as in monocytes [16]. The TF activates the extrinsic blood coagulation cascade [6,17]. When a vascular injury occurs, the subendothelial TF is exposed to the blood flow and binds the plasma factor VIIa. The TF is the factor VII receptor and forms the complex TF-VIIa which initiates the coagulation by activating the zymogens factor X and IX to their respective serine proteases, factor IXa and factor Xa. Factor IXa and Xa form complex enzymes with their nonenzymatic co-factors (factor VIIIa and factor Va) on the surface of the membranes containing acidic phospholipids. Altogether, this leads to the thrombin generation from the zymogen prothrombin (Figure 1). Subsequently, thrombin activates fibrinogen in order to form fibrin, which leads to the clot formation [17,18]. Moreover, thrombin can stimulate its own production. The complex formed by the TF and factor VIIa (TF-VIIa) is essential for normal hemostasis [16,19,20,21]. Habitually, cells in contact with blood do not express a physiologically active TF and the exposure of the TF to blood is the critical cause for most of its functions [22]. The TF is strategically located in cells underneath the endothelium of the vasculature, which allows for its rapid recruitment in response to chemical or physical damage. The inflammatory cytokines (TNFα and IL-1β), infectious agents, and other injuries can upregulate the TF expression, and it will become exposed to the circulation.

As an additional regulatory mechanism, the TF is produced in an inactive form that becomes activated following several poorly understood mechanisms: the dimer formation, lipid raft, disulfide bonds, or phosphatidylserine exposure. Phosphatidylserine is a glycerophospholipid that participates in the activation of several key signaling pathways, such as the coagulation pathway. Still, it is not clear how the TF activation works, but there may be more than one mechanism acting simultaneously. For example, the exposure of phosphatidylserine to the cell surface seems to be able to trigger the formation of activated factors Va, IXa, VIIIa, and Xa, which will contribute to the correct orientation of the TF-VII complex [17,23]. However, the contribution to coagulation by the TF and phosphatidylserine is different; the TF appears to be the physiological activator, and phosphatidylserine is the driving force behind the propagation of coagulation.

The activation of factors VIIa, Xa, and thrombin activates the platelets and induces the protease-activated receptor (PAR) -mediated signaling, which leads to the release of pro-inflammatory cytokines and upregulates the expression of the leukocyte and vascular adhesion molecules (ICAM-1 and VCAM-1).

The TF may cause the activation of damage-associated molecular patterns (DAMPs) that are generated during infection, inflammation, or stress. Consequently, it triggers the activation of toll-like receptors (TLRs) mostly upon immune cells and the complement system, which induces the release of pro-inflammatory cytokines and chemokines, the upregulation of leukocyte adhesion molecules, and the expression of the TF.

### 2.2. Thrombin and PARs

Thrombin cleaves fibrinogen and generates a fibrin clot (Figure 1). Thrombin has a positive feedback loop through a pro-cofactor activation that increases its own production. Moreover, it can activate other cell types via the cleavage of the PARs. The PARs are primarily expressed in platelets and endothelial, immune, and epithelial cells. The PAR activation triggers the production of the monocyte chemoattractant protein-1, TNFα, IL-1β, and IL-6.

The PAR signaling in the endothelial cells causes their activation with an increase in the P- and E-selectin expression, as well as the increase in MCP1/CCL2, IL8, and the plasminogen activator inhibitor 1 (PAI-1). Altogether, this triggers the leukocyte and platelet recruitment and the adhesion to the endothelium, thereby increasing the risk of thrombosis. Overall, this process is a powerful positive feedback loop that intensifies the inflammation and pro-coagulant processes.

### 2.3. Protein C–Thrombomodulin–Endothelial Cell Protein C Receptor

Protein C (PC) is a precursor for the activated protein C (APC), which is a serine protease necessary to prevent the dysregulated activation of the coagulation and inflammation. The APC generation depends on the cleavage by thrombin and is catalyzed by thrombomodulin (TM) and the endothelial protein C receptor (EPCR). It has been described that the APC is able to suppress the nuclear translocation of NF-κB. Therefore, the APC reduces the release of the pro-inflammatory cytokines, including TNFα, IL-1β, IL-6, IL-8, and the macrophage inflammatory protein-1α by the monocytes/macrophages, it diminishes the TF, and inhibits the neutrophil activation and chemotaxis, thereby reducing inflammation.

### 2.4. Toll-Like Receptors

Toll-Like-Receptors (TLRs) are expressed in a broad number of cells such as the endothelial cells, platelets, macrophages, and dendritic cells and these play a major role in regulating the innate immune system and have the capacity to trigger several inflammatory pathways [24]. The TLRs may contribute to thrombosis, but the mechanisms are not fully understood. Several pre-clinical models of diabetes, hypercholesterolemia, and endotoxemia have shown that TLR2 and TLR4 mediate the TF expression and the platelet activation, thereby increasing the coagulation response [25,26,27,28]. Fibrinogen may act as a ligand for the TLR-mediated signaling. The signaling through TLR9 in human coronary artery endothelial cells and pre-clinical mouse models are involved in initiating the TF expression [29].

### 2.5. Complement System

The complement system is essential for the innate immune system response. The complement system is activated via the classical, lectin, or alternative pathways [30,31]. These three pathways converge at the C3 complement node, which is a pivotal protein. In addition, the complement system contributes to the inflammation and thrombosis [32,33]. Mainly, the complement enzymes comprise a single serine protease with a high substrate specificity. However, it has been shown that the complement and coagulation pathways share some serine-proteases that may be shared by activating at the same time factor (F) XIIa, which is able to activate C1q [34].

C5a was generated in the absence of C3, with thrombin acting as a potent C5 convertase [35]. The complement system also can amplify the coagulation through the C5a-mediated induction of the expression of the TF in neutrophils, endothelial cells, monocytes [36,37], plasminogen-activator inhibitor 1 (PAI-1), and on the von Willebrand factor (VWF) secretion from the endothelial cells.

### 2.6. Plasminogen System and Fibrinolysis

The fibrinolytic system is responsible for the resolution of blood clots; nonetheless, this system can also trigger the inflammatory processes involved in thrombosis [38]. The plasminogen system leads to fibrinolysis by progressively degrading fibrin which initiates several immune processes that help wound healing and angiogenesis [39]. The fibrinolytic system is activated directly upon the fibrin formation.

Thrombin produced during hemostasis or thrombosis is able to activate the endothelium and produce and secrete the plasminogen activators and the urokinase type-plasminogen activator (uPA). The plasminogen activators and the uPA cleave the plasminogen in order to generate plasmin, which is the principal fibrinolysis enzyme. Fibrin facilitates the interaction between the tissue plasminogen activator (tPA) and the plasminogen, thereby acting as a cofactor for the tPA-mediated plasminogen activation. Moreover, the cellular and fibrin-based plasmin generation can activate C3 and C5 of the complement system, thereby mediating the inflammation [40].

### 2.7. Platelets and Clot Formation

The platelet and coagulation activations are interconnected processes. Platelets are one of the key players in clot formation [41]. Collagen and thrombin via the TF initiate the arterial thrombus formation. At the initial stages of the process, the coagulation factors bind to the membrane receptors on resting platelets, which initiate the platelet adhesion and thrombus formation. When platelets become active, they express more binding sites a with high affinity to the activated coagulation factors. The activated platelets cluster together, they release their content, and form a clot. The released content, mainly thrombin, can activate other platelets and interact with the pro-coagulation factors that respond in a cascade, thereby converting fibrinogen, a blood-soluble protein, into fibrin that is the end coagulation product and that lays down into the clot and strengthens it.

## 3. Mesenchymal Stromal Cells (MSCs) Modulate Coagulation

The MSCs can regulate the coagulation in a wide range of pathways. Here, we will review the mechanisms that have been fully confirmed and summarize other non-well-understood possible pathways.

### 3.1. TF-Mediated Pro-Coagulant Activity of the MSCs

Growing evidence is emerging concerning the pro-coagulant effect of MSCs through the release of the tissue factor (TF) [5,6,13,14,42] which could increase the risk of clot formation. MSCs express the surface tissue factor (TF) and can also release it in the microenvironment. Several inflammatory mediators can trigger the expression of the TF by the MSCs [43]. Moll et al. first described that the increased passaging of MSCs during culture leading to a remarkable increase in the expression of the TF on the cell surface of the MSCs [44]. This was also independently verified by Tatsumi and Liao in subsequent studies that further discussed the tissue sources from which the cells were derived [45,46]. Recently, Moll et al. summarized the occurrence of the adverse thrombotic reactions upon the infusion of the TF expressing MSCs in patients [5]. The intrinsic blood-activating properties of MSCs, depending on their source, have been studied by several authors who have discussed how to control the activation of the clotting system [47,48].

As mentioned in the previous chapter, the TF triggers the extrinsic blood coagulation cascade by forming the complex TF:VIIa, followed by the activation of factors X and IX, thereby leading to the thrombin formation from the zymogen prothrombin [6,17]. The cultured MSCs, once they are exposed to blood, generate an immune response called the instant mediated blood mediated inflammatory response (IBMIR) that complements the coagulation activation together with the activation of the leukocyte and platelet populations; in 2012, Moll et al. reported for first time about the IBMIR, in relation to clinical MSC products [44,49]. This response was initiated by the increased formation of thrombin and clotting factors, such as activated factors VIIa, XIa, and XIIa. A clear dose-dependent correlation has been described between the MSC-associated TF with a clot formation; a higher TF density measured by flow cytometry was associated with a shorter clotting time and a greater thrombin production [23]. Different MSC tissue sources display highly variable expressions of the TF. For example, it has been shown that isolated umbilical cord (UC), bone marrow (BM), and adipose tissue (AT) derived MSCs express the TF in cell culture conditions. However, the BM-MSC expresses lower levels of the TF than the AT-MSC or the UC-MSC [48,50]; the BM-MSC showed a much lower pro-coagulant activity in vitro; meanwhile, the AT-MSC exhibited a high pro-coagulant activity.

In vivo, the administration of the low TF-expressing BM-MSCs in rats did not show any intravascular clot formation; meanwhile, the administration of the TF-expressing UC-MSC triggered an intravascular thromboembolism in the lungs, liver, and spleen [48]. Likewise, it has been reported that the TF-expressing BM-MSC intravenous administration in mice spreads the micro-thrombi in the heart, kidney, spleen, and liver [46]; that effect could be prevented by the administration of heparin (400 U/kg). Consequently, as the AT-MSCs seems more consistently pro-coagulant than the BM-MSCs, they are presenting a potential safety concern for the systemic administration in coagulopathic patients.

While a considerable number of in vitro and pre-clinical studies have confirmed that MSCs exert a pro-coagulant effect after blood contact, it is clear that in clinical studies, the adverse events related to thrombosis exist, however they are underreported and these limited studies report these thrombotic events in humans associated with an MSC administration [51]; here we summarize them. The intravenous administration of MSCs in 44 patients, who underwent a hematopoietic stem cell transplantation and who suffered from a weak IBMIR, the response was highly variable depending on the donor, the number of administered cells, and their cellular passage [44]. Moreover, the cell cryopreservation and freeze-thawing effects should also be considered; the IBMIR was slightly augmented in the thawed cells, likely due to the release of the intracellular TF that may increase clotting and trigger a complement activation [52,53,54]. Moll et al. found that the patients exhibited an increased formation of the blood activation markers but no formation of the hyperfibrinolysis marker D-dimer [44]. In Phase 1b/2a of the clinical study into Crohn’s disease, Melmed et al. reported two patients suffering from venous thrombosis after the infusion of a placenta isolated high-TF-expressing MSC. They associated the adverse effect with the TF expression as a cause [6,14,44,55]. A man who received multiple systemic AT-MSC infusions for a herniated cervical disc, presented with a bilateral pulmonary embolus one month after receiving the last one [56]. Another 73-year-old man enrolled in a clinical trial died from a pulmonary embolism after receiving an infusion of AT-MSCs [57]. However, both studies do not describe the cell source, the manufacturing process, or the internal control of the administered cells. Numerous clinical trials using MSC therapies are in progress, and the TF expression may arise as a safety principle. Furthermore, many patients who may benefit from MSC therapy are likely to be in a hypercoagulable state or at a high risk of a thrombotic event due to their primary pathology. Lalu et al. reported, in their systematic review and meta-analysis, that MSC therapy appears to be safe based on the current clinical trials, nevertheless they mention that further larger scale controlled clinical trials are required in order to further define the safety profile of the MSCs [58]. Therefore, monitoring the pro-thrombotic effects of the MSCs during manufacture in order to ensure that they show the desired characteristics prior to patient administration should be part of the evaluated criteria in clinical trials. Moll et al. highlight, in several of their most recent publications, the clear need for new and improved clinical guidelines regarding the usage of MSC-derived therapeutics [5,6,13].

The TF is not the unique pro-coagulation factor expressed by MSCs; MSCs also express phosphatidylserine, which, after moving from the cytoplasm to the cell surface (membrane), can trigger the thrombin-activating complex and thereby activate coagulation [59,60].

Moreover, an preliminary in vitro study that confirmed that cultured fibrin-embedded MSCs express the plasminogen activators uPA, tPA, and PAI [61], thereby suggesting that MSCs are also involved in the fibrinolytic cascade. In vivo, the autologous AT-MSCs administration induced peripheral micro thrombosis in 30 diabetic patients (type 1 and 2) with limb ischemia [62]. Furthermore, the AT-MSCs derived from the type 2 diabetic patients exhibited a decreased serum-independent fibrinolytic activity. Later studies demonstrated that the AT-MSCs isolated from the diabetic patients released greater amounts of PAI-1, less tPA, and formed fewer D-dimers compared with the MSCs isolated from healthy patients. In addition, the diabetic AT-MSCs presented an upregulation of the expression of the TF and displayed an altered signaling of platelet-derived growth factor (PDGF) [63]. Consequently, MSCs seem to play a dual role by provoking the IBMIR explained by the TF secretion and by producing fibrinolytic enzymes that can activate the fibrinolytic cascade.

### 3.2. Effect of MSCs in Platelet Formation

MSCs can play a role in coagulation by acting directly in the platelet production and activation. Kim et al. have shown that the co-administration of MSCs together with a bone marrow transplantation (BMT) can trigger a platelet production, thereby enhancing coagulation. These results suggest that MSCs can be therapeutic in preventing thrombocytopenia after the BMT by promoting megakaryopoiesis and platelet differentiation [64].

In agreement, Liao et al. described that the BM-MSC infusion decreased platelet numbers in blood in a concentration-dependent manner [46]. The infusion of more than 1 × 106 MSCs prolonged the prothrombin time (PT), activated the partial thromboplastin time (APTT), and decreased fibrinogen and factor VIII concentrations. The increased numbers of the MSC administration were associated with acute adverse events, including an IBMIR [44] and microvascular embolisms [65,66,67]. The infusion of a small dose of MSCs affected the PT but not the APTT, thereby suggesting that the main activation of coagulation occurred through the TF and the extrinsic coagulation pathway. The administration of a TFPI was able to prevent an MSC-induced coagulation. The pre-injection of the heparin also prevented the MSC-induced coagulation reaction, which prevented the circulatory and respiratory failure produced for MSCs in higher doses.

### 3.3. MSC Released Soluble Factors Affecting Coagulation

The MSC-conditioned medium was tested for platelet activation in order to demonstrate whether they released any soluble factor that could trigger clot formation and enhance coagulation. The MSC-conditioned medium did not affect the platelet activation, in neither the resting platelets nor in the stimulated ones. The conditioned medium obtained from the MSC culturing slightly suppressed the expression of CD62p and Pac-1, which are two platelet activation markers.

Moreover, the extravesicles (EVs) secreted by MSCs were also analyzed. The TF was not detected in the EVs, which supports that the MSC-induced coagulation may be TF-mediated, but the TF is not a relevant factor in the pro-coagulant effect of the EVs. Previously, it has been shown that the monocyte and platelet-secreted EVs trigger a thrombin generation and propagation of the coagulation through the exposure of phosphatidylserine, and the TF was only detected in pathological conditions [68]. It has been described that the MSC-derived EVs can expose phosphatidylserine onto their surface [69].Phosphatidylserine offers a binding, the catalytic point that leads to the formation of factors Va, VIIIa, Ixa, X, and Xa and to the activation of the TF.

We may speculate that the coagulation activity of the EVs derived from MSCs is associated with the activation of the coagulation cascade by phosphatidylserine rather than the TF [59,70]. Silachev et al. revealed that only a part of the MSC and EV populations (3–4%) were annexin V-positive and presented phosphatidylserine on their surface; the authors confirmed the phosphatidylserine involvement in the pro-coagulant effects caused by MSCs and their EVs [60].

## 4. Conclusions

The MSC-associated coagulation effects have been described and studied; however, a lot of research is still needed in order to better determine which cell source for obtaining MSCs is the best in order to avoid a pro-thrombotic response due to their infusion. In addition, in order to characterize the MSC expression markers such as the TF, we need to control the cell dose, the culture passage, and the administration route, which may help to reduce and avoid adverse coagulation events triggered by MSC therapies.
